# Prognostic value of physicians' assessment of compliance regarding all-cause mortality in patients with type 2 diabetes: primary care follow-up study

**DOI:** 10.1186/1471-2296-7-42

**Published:** 2006-07-07

**Authors:** Dietrich Rothenbacher, Gernot Rüter, Hermann Brenner

**Affiliations:** 1 Dept. of Epidemiology, German Centre for Research on Ageing, Heidelberg, Germany; 2 Div. of Clinical Epidemiology and Aging Research, German Cancer Research Center, Heidelberg, Germany; 3 General Practitioner; Benningen/Neckar, Germany

## Abstract

**Background:**

Whether the primary care physician's assessment of patient compliance is a valuable prognostic marker to identify patients who are at increased risk of death, or merely reflects measurement of various treatment parameters such as HbA_1C _or other laboratory markers is unclear. The objective of this prospective cohort study was to investigate the prognostic value of the physicians' assessment of patient compliance and other factors with respect to all-cause mortality during a one year follow-up period.

**Methods:**

A prospective cohort study was conducted among 1014 patients with type 2 diabetes aged 40 and over (mean age 69 years, SD 10.4, 45% male) who were under medical treatment in 11 participating practices of family physicians and internists working in primary care in a defined region in South Germany between April and June 2000. Baseline data were gathered from patients and physicians by standardized questionnaire. The physician's assessment of patient compliance was assessed by means of a 4-point Likert scale (very good, rather good, rather bad, very bad). In addition, we carried out a survey among physicians by means of a questionnaire to find out which aspects for the assessment of patient compliance were of importance to make this assessment. Active follow-up of patients was conducted after one year to determine mortality.

**Results:**

During the one year follow-up 48 (4.7%) of the 1014 patients died. Among other factors such as patient type (patients presenting at office, nursing home or visited patients), gender, age and a history of macrovascular disease, the physician's assessment of patient compliance was an important predictor of all-cause mortality. Patients whose compliance was assessed by the physician as "very bad" (6%) were significantly more likely to die during follow-up (OR = 2.67, 95% CI 1.02–6.97) after multivariable adjustment compared to patients whose compliance was assessed as "rather good" (45%) or "very good" (18%). The HbA_1C_-value and the cholesterol level at baseline showed no statistically significant association with all-cause mortality. According to our survey for most of the physicians self-acceptance of disease, treatment adherence, patient's interest in physician's explanations, attendance at appointments, a good self-management, and a good physician-patient relationship were key elements in the assessment of patient compliance.

**Conclusion:**

The primary care physician's assessment of patient compliance is a valuable prognostic marker for mortality among patients with type 2 diabetes. Identification of patients in need of improved compliance may help to target preventive measures.

## Background

Type 2 diabetes mellitus (DM) is the most common endocrine disease in developed countries and prevalence is increasing worldwide [1]. In Germany, about 5% of the population are affected and the prevalence is increasing dramatically with age [2]. Patients with type 2 diabetes have an approximately threefold risk for all cardiovascular diseases [3,4] and their risk of death from all causes is increased by 75% compared to patients without diabetes. The morbidity and mortality resulting from complications of type 2 diabetes such as micro- and macrovascular disease cause considerable costs for the individual and the society [2,5] and necessitate special care and attention [6].

It is well known that the likelihood of diabetic complications, and subsequently risk to die, in patients with diabetes is associated with glycemic control [7-11] and adherence to treatment and successful self-management. Whether the physicians' assessment of patient compliance is a valuable prognostic information to identify patients who are at increased risk for subsequent complication or death, or merely reflects measurement of various treatment parameters such as HbA_1C _or other laboratory markers is unclear yet. However, as most patients are treated in primary care setting, this information would be critical and would eventually allow to identify subjects in need for improved diabetic therapy and help to target preventive measures.

The objective of this prospective cohort study including all patients with type 2 diabetes who were under medical treatment in the primary care practices of family physicians and internists in a geographically defined region was to investigate various aspects and the prognostic value of the physicians' assessment of the patient compliance with respect to all-cause mortality during a one year follow-up period.

## Methods

### Study design and study population

We included all patients with known DM type 2, aged 40 and over in a longitudinal study, who were under medical treatment in one of 11 participating practices of general practitioners (general practitioners or internists working in primary care in a coherent region in South Germany) between April and June 2000. Some surgeries were shared by 2 GPs, in total 15 physicians were involved. In addition to the patients who presented themselves in the surgeries of the general practices we also included all patients who were seen in care homes or visited at home by the participating GPs. DM was defined as having ever fulfilled the following diagnostic criteria: nonfasting blood glucose of > 200 md/dl or a fasting glucose of ≥ 126 mg/dl according to the diagnosic criteria of the American Diabetes Association.

### Data collection

This study was part of the quality assurance program of the participating practices. All data collection procedures and diagnostic criteria were defined in a manual and all practice teams were trained accordingly before study conduct. All patients who presented at the surgeries of the GPs were asked to fill out a standardized questionnaire which contained information about medical history, life style habits, and health related quality of life. The physicians filled out a separate questionnaire with details to diagnosis of DM, treatment, existing comorbidity (as documented in the patient's charts), most recent laboratory results and other patient related information for patients presenting at the surgeries as well as for the patients who were seen in nursing homes or visited at home. Participation was voluntary and all information was collected anonymously at the time of the patient's office visit. The informed consent of the patient was documented on the physician's questionnaire. The study has been carried out in compliance with the Helsinki Declaration.

Each physician was asked to assess the compliance of each patient by the following question: "How would you assess the compliance of this patient?" The answer was asked on a four point Likert scale (very good, rather good, rather bad, very bad). In addition, after all patients had been included, we carried out a qualitative survey among physicians by means of a standardized questionnaire to find out which aspects for the assessment of patient compliance were of strong or very strong importance to make this assessment. The physicians were asked at the end of the study what meaning the following ten statements had for them in general when answering the question related to patients' compliance: 1) "The patient seems to have no self-acceptance of his disease"; 2) "The patient shows no interest in my explanations and councelings"; 3) "The patient keeps re-appointments only sporadicly"; 4) "The patient is adherent to the recommended treatment"; 5) "The patient has poor social or family support"; 6) The patient often has a high HbA_1C_-value"; 7) "The patient has a good self-management"; 8) "The patient has many other concurrent diseases"; 9) "The patient has a bad health-related quality of life"; 10) "The physician-patient relationship is bad". For every question the following response was possible on a four-point Likert scale ("very important meaning, rather important meaning, rather less important meaning, no importance").

HbA_1C _of all participating patients was measured as part of the routine medical care in the same laboratory by means of High Performance Liquid Chromatographie (HPLC) on a Tosoh HLC 723 and total cholesterol concentrations were determined by routine enzymatic methods.

One year after the inclusion in this study, patients' mortality was recorded by the 11 practices. For all 1014 included patients information on mortality (dead/alive at follow-up) could be obtained.

### Statistical analysis

The study population is presented in a descriptive way. In addition, we determined the association of various personal, sociodemographic, life style, medical and laboratory characteristics (a HbA_1C _value ≥ 8%, a total cholesterol value of ≥ 257 mg/dl (≥ 8^th ^percentile)), and of physician's assessment of patient compliance with all-cause mortality and used a χ^2^-test to assess the significance of the associations.

We then used multivariable logistic regression with a backward variable selection procedure to identify the independent association of various factors with all-cause mortality. The following variables were considered for the initial model: gender (male, female), patient type (patients presenting at office, nursing home or visited patients), age (decades), family status (single, married, widowed, divorced), time since diagnosis of diabetes (< 1 year, 1 to 4 years, 5 to 10 years, > 10 years), macrovascular complications (myocardial infarction, apoplex, or pheripheral arterial vascular disease), microvascular complications (retinopathy, nephropathy, latter defined by microalbuminuria (neuropathological complications not included as they were difficult to assess in the context of this study in primary care)), HbA1_C_-value (continuous), total cholesterol (continuous), prescription of ACE-medication or lipid-lowering drugs, the blood pressure values from baseline, patient compliance (physicians' assessment) (very good, rather good, rather bad, very bad).

We then eliminated step by step all variables which did not contribute to the model in a statistically significant way (p > 0.1). Odds ratios (OR) and 95% confidence intervals were calculated for all variables included in the final model.

## Results

In total, 1014 patients with known DM aged 40 and over were included in this study. Response among all eligible patients with DM was 88%. 882 (87%) of the patients presented themselves in the 11 participating general practices and 132 (13%) were seen in nursing homes or visited at home by the physicians.

As table 1 shows, 55% of the patients with DM were women. Mean age of patients with DM was 69.0 years (SD 10.4, range 40 to 91 years), two thirds of them (66%) were between 60 and 80 years old, and 65% were married. The time since diagnosis was less than 5 years among 34% of patients, 5 to 10 years among 32% of patients, and more than 10 years among 30% of patients. The physicians' assessment of patient compliance was in general more favourable for the patients presenting at the surgery. Overall, 18% of patients were judged as "very good", 45% as "rather good", 31% as "rather bad", and 6% as "very bad" regarding their compliance.

**Table 1:** Sociodemographic and medical characteristics of study population at baseline

	**Patients presenting at office**		**Care home or visited patients**		**All**	
*n*		*882*		*132*		*1014*
	n^a^	%	n^a^	%	n^a^	%

**Gender**						
- Male	424	48	34	25	458	45
- Female	453	52	98	75	551	55
**Age (years)**						
Mean (SD)	67.3 (9.6)		80.1 (8.6)		69.0 (10.4)	
- 40 – 49	38	4	1	1	39	4
- 50 – 59	173	20	2	2	175	17
- 60 – 69	331	38	11	8	342	34
- 70 – 79	272	31	52	39	324	32
- 80 +	65	7	66	50	131	13
**Family status**						
- single	36	4	14	11	50	5
- married	593	72	34	26	627	65
- widowed	168	20	78	59	246	26
- divorced	30	4	5	4	30	4
**Time since diagnosis of Diabetes (years)**						
< 1 year	68	8	3	2	71	7
1 to 4 years	246	28	24	19	270	27
5 to 10 years	288	33	40	30	328	32
> 10 years	246	28	59	44	305	30
unknown	34	4	6	5	40	4
**Diabetes medication**						
- Diet only	282	32	48	36	330	32
- Medication (Combinations possible)						
- Insulin	194	22	50	38	244	25
- Sulfonylureas	332	38	38	29	370	36
- Metformin	304	34	14	11	318	31
**Patient compliance (physicians' assessment)**						
- very good	164	19	11	9	175	18
- rather good	403	46	48	38	451	45
- rather bad	255	29	52	41	307	31
- very bad	46	5	15	12	61	6

^a ^numbers do not always add up to total due to missing information in few items

During the one year follow-up period 48 (4.7%) of the patients died. Table 2 shows the all-cause mortality according to various factors. Mortality was much higher in patients visited in nursing homes or at home compared to patients presenting at the surgery (18.2% vs. 2.7%. In bivariate analysis gender was not associated with all cause mortality. However, mortality strongly increased with age and was highest in widowed subjects. Time since diagnosis of DM, and presence of macrovascular or microvascular complications at baseline were clearly associated with a higher proportion of subjects who died during follow-up.

**Table 2:** All cause mortality according to various sociodemographic and medical factors

	**Died during follow-up**		
		
	(n/N)^a^	%	p-value
**Total**	48/1014	4.7	
**Patient type**			
- Patients presenting at office	24/882	2.7	
- Care home or visited patients	24/132	18.2	< 0.0001
**Gender**			
- Male	26/458	5.7	
- Female	22/551	4.0	0.2
**Age (years)**			
- 40 – 59	1/214	4.7	
- 60 – 69	9/342	2.6	
- 70 – 79	16/324	4.9	
- 80 +	22/131	16.8	< 0.0001^b^
**Family status**			
- single	4/50	8.0	
- married	22/626	3.5	
- widowed	22/246	8.9	
- divorced	0/34	0	0.004^b^
**Time since diagnosis of diabetes (years)**			
- < 1 year	0/71	0	
- 1 to 4 years	9/270	3.3	
- 5 to 10 years	17/328	5.2	
- > 10 years	21/305	6.9	0.04^b^
**Macrovascular complications at baseline**			
- no	12/625	1.9	
- yes	36/398	9.3	< 0.0001
**Microvascular complications at baseline**^c^			
- no	25/718	3.5	
- yes	23/296	7.8	0.005

^a ^numbers do not always add up to total due to missing information in few items

^b ^Fisher's Exact Test

^c ^(neuropathological complications not included as difficult to assess in the context of this study in primary care)

The physicians' assessment of patient compliance during baseline was also associated with an increased mortality (**see **table 3). Patients who's compliance was judged at baseline as "very bad" showed a 1-year mortality of 13.1% compared to a 1-year mortality of 4.2% ("rather bad"), 3.8% ("rather good"), and 4.0% ("very good") in the respective categories (p = 0.01). By contrast, a high HbA_1C_-value (≥ 8%) and a cholesterol value ≥ 257 mg/dl (≥ 8^th ^percentile) at baseline showed no statistically significant association with all-cause mortality during the one year follow-up (**see **table 3).

**Table 3:** All cause mortality according to physicians' assessment of patient's compliance and various laboratory markers at baseline

	**Died during follow-up**		
		
	(n/N)^a^	%	p-value
**Total**	48/1014	4.7	
**Patient compliance (physicians' assessment)**			
- very good	7/175	4.0	
- rather good	17/451	3.8	
- rather bad	13/307	4.2	
- very bad	8/61	13.1	0.01
**HbA1**_C_**-value**			
- < 8%	32/766	4.2	
- ≥ 8%	12/208	5.8	0.32
**Total cholesterol**			
- < 257 mg/dl	40/789	3.0	
- ≥ 257 mg/dl (≥ 80^th ^percentile)	6/199	5.1	0.22

^a ^numbers do not always add up to total due to missing information in few items

We then used unconditional logistic regression and a stepwise backward selection procedure to find the most parsimonious model of factors associated with all-cause-mortality (**see **table 4). Patients seen at nursing homes or visited at home had a higher odds to die within the follow-up period compared to patients presenting at the surgery (OR 3.92, 95% CI 1.85–8.30). In addition, female patients showed significantly lower odds to die compared to males (OR 0.34; 95% CI 0.17–0.67). In addition, there was a clear increased risk to die with increasing age. Furthermore, the presence of macrovascular complications was a statistically significant determinant of subsequent risk to die (OR 3.17, 95% CI 1.57–6.39). The physicians' assessment of the patient compliance was also an important factor associated with all-cause mortality. Patients whose compliance was assessed as "very bad" had an OR of 2.67 (95% 1.02–6.97) compared to patients assessed as "very good" or "rather good". The fit of the model did not improve and the observed association did not change if HbA_1C_-values, total cholesterol, prescription of ACE-medication or lipid-lowering drugs, or the blood pressure values from baseline were added in the model (data not shown). None of the latter four variables met the criteria for inclusion in the model.

**Table 4:** Variables found to be independently associated with all-cause mortality: results of multivariable logistic regression (all variables controlled for each other)

**Characteristic**	**Odds ratio and (95% CI) controlled for covariates**	**p-value**
**Patient compliance (physicians' assessment)**		
- very good or rather good	1^Reference^	
- rather bad	0.78 (0.38–1.61)	0.50
- very bad	2.67 (1.02–6.97)	0.04
**Patient type**		
- Patients presenting at office	1^Reference^	
- Nursing home or visited patients	3.92 (1.85–8.30)	0.0004
**Gender**		
- Male	1^Reference^	
- Female	0.34 (0.17–0.67)	0.002
**Age (years)**		
- 40 – 59	1^Reference^	
- 60 – 69	4.79 (0.59–38.67)	0.14
- 70 – 79	7.98 (1.01–63.16)	0.05
- 80 +	20.46 (2.47–169.45)	0.005
**Macrovascular complications**		
- no	1^Reference^	
- yes	3.17 (1.57–6.39)	0.001

Finally, we carried out a small survey among the fifteen physicians to find out which aspects of compliance were most important for them when making this assessment on a individual basis. For most of them self-acceptance of disease, treatment adherence, patient's interest in physician's explanations, attendance at appointments, a good self-management, and a good physician-patient relationship were key elements. Patient's co-morbidity, a high HbA_1C _value, a good social support, and quality of health related life were other, sometimes less important elements in their decision making.

## Discussion

In this study physicians' assessment of patient compliance was found to have an independent prognostic value for subsequent risk of death, which persisted after control for other factors. Compliance may reflect many aspects of successful self-management of diabetes and the identification of patients in need of improved compliance may help to target preventive measures.

It is well accepted that an optimal glucose control, adherence to a treatment regimen, and successful self-management minimizes the likelihood of diabetic complications, and subsequently, the risk of death [7-11]. However, many different components seem to be related to successful self-management of this chronic disease; provider characteristics, the patient-physician relationship, and patients' personal characteristics and beliefs, as well as environmental, social, and cultural factors act as important contributors to this complex issue [12]. Although physicians' assessment of patient compliance appears to be strongly related with glycemic control [13], its prognostic value was found to be independent of glycemic control in this study and prognostically more relevant than gylcemic control.

The key element in management of chronic diseases is self-management. Unfortunately, many patients are not willing or able to take full responsibility for their care [14]. Successful treatment should be regarded as a team approach, incorporating patients, care givers and family members and the social context of the individual [15]. Factors such as a good team spirit in medical practice can improve management of patients with chronic diseases [16]. However, attempts to increase the frequency of appointments or the use of other measures in patients with poor compliance may be counterproductive. Many of these patients will react with even more resistance. A promising approach may be to strengthen the "sense of coherence" by taking aspects of comprehensibility, manageability and meaningfulness from the patients' social and personal perspective into account [17]. As earlier work suggested individual differences in perceived control and coping strategies may strongly interact with management of chronic diseases [18]. However, clearly more studies especially in primary care setting are needed to further determine which factors pose obstacles to an integrative conception of a successful treatment of chronic diseases and what compliance means from the physician's as well as the patient's view. As shown by the cross-national Diabetes Attitudes, Wishes and Needs (DAWN) study, psychosocial problems in patients with diabetes are common according to reports of patients and providers, and are perceived as important barriers to effective self-care. The regimen adherence of patients was less than optimal in all ten participating countries (Germany included) and the providers believed that mainly psychological problems were the cause. However, providers also reported that they often do not have the critical resources for addressing these problems [19].

If looking at the results of this study the following strengths and weaknesses should be considered: Relatively few patients were assessed as having a "very bad" compliance and only for this group the prognostic relevance was evident. Unfortunately, we had no comparison with another related sample or norm groups. Furthermore, we did not measure a specific component of compliance such as treatment adherence (e.g. pills counts) or the compliance with a special diet. However, we measured physicians' assessment of the patient compliance as an integrative measure (including also aspects of self-management behaviour) and from our small qualitative survey among the physicians we got important clues which aspects of compliance were most important for them when making this assessment on an individual basis. Notably, outcome parameters such as HbA_1C_, the social network, and the health related quality of life were less important elements in their decision making with respect to assessment of patient compliance.

This study was conducted in the framework of a quality assurance program of the participating practices including all patients within a defined geographic region, irrespective of their insurance affiliation. As in Germany almost all patients have access to health care, as all primary care physicians within a coherent region participated, and as most patients with DM usually consult their primary care giver once in three months, we are confident that the study population comprised almost all patients with type 2 DM in primary medical care in the study region. However, patients not seeking medical care and patients hospitalized were certainly underrepresented in this study.

## Conclusion

Having the aforementioned caveats in mind, our study suggests that the primary care physician's assessment of patient compliance is a valuable prognostic marker for the risk of subsequent death among patients with type 2 diabetes. Identification of patients in need of efforts to improve compliance may help to target preventive measures.

## Abbreviations

CI = confidence interval

DM = diabetes mellitus

GPs = general practitioners

HbA_1C _= hemoglobin A_1C_

OR = Odds ratio

## Competing interests

The author(s) declare that they have no competing interests.

## Authors' contributions

DR did the statistical analysis and wrote the initial draft. HB, GR and DR had the idea of the study and HB, GR and DR did study design and conduct. All authors critically revised the MS and read and approved the final manuscript

## Pre-publication history

The pre-publication history for this paper can be accessed here:


